# Infancy and Childhood Obesity Grade Predicts Weight Loss in Adulthood: The ONTIME Study

**DOI:** 10.3390/nu13072132

**Published:** 2021-06-22

**Authors:** Eva Morales, Nathaly Torres-Castillo, Marta Garaulet

**Affiliations:** 1 Biomedical Research Institute of Murcia (IMIB-Arrixaca), 30120 Murcia, Spain; evamorales@um.es; 2 Department of Public Health Sciences, University of Murcia, 30100 Murcia, Spain; 3 Department of Molecular Biology and Genomics, Institute for Translational Nutrigenetics and Nutrigenomics, Health Sciences University Center, University of Guadalajara, Guadalajara 44340, Jalisco, Mexico; nathaly.torres.c@gmail.com; 4 Department of Physiology, Regional Campus of International Excellence, University of Murcia, 30100 Murcia, Spain; 5 Division of Sleep and Circadian Disorders, Brigham and Women’s Hospital, and Division of Sleep Medicine, Harvard Medical School, Boston, MA 02115, USA

**Keywords:** intergenerational obesity, size at birth, body mass index, weight loss, dietary treatment

## Abstract

We examined the relationships between intergenerational obesity, weight and size at birth, and obesity from infancy to adolescence with weight loss in response to a dietary intervention. We studied 4264 participants (3369 women; mean age 41.5 ± 12.9 years) of the ONTIME study. Participants followed a weight-loss treatment based on a Mediterranean diet. Associations between grandparental and parental obesity grade, birth weight and size, and obesity grade in infancy, childhood and adolescence with total weight loss in response to treatment were assessed, using multivariate linear regression models. A lower weight loss (kg) in response to treatment was found among participants who were obese during infancy (beta coefficient −2.13 kg; 95% CI, −3.96, −0.30; *p* = 0.023). Furthermore, obesity during infancy and also during childhood was associated with a slower weekly rate of weight loss during treatment (*p* < 0.05). In conclusion, obesity in infancy and in childhood impairs the weight-loss response to dietary treatments in adulthood. Tackling obesity throughout early life may improve the effectiveness of weight-loss interventions in adulthood.

## 1. Introduction

Obesity represents an important public health concern. It contributes notably to the overall burden of disease, mortality, and quality of life [1]. Currently, the prevention and improvement of interventions remain crucial to tackle the global obesity epidemic.

Disentangling factors that influence the effectiveness of dietary treatments for obesity in clinical practice is still challenging [2]. A high interindividual variation in weight-loss treatment effectiveness is common, and diverse types of barriers are involved [3]. It is likely that these barriers may include not only physical, environmental, and emotional factors but also an endogenous predisposition of individuals. In this regard, the individual ability to lose weight in response to treatments could be influenced not only by genetic factors [4] but also be programmed by an adverse development during the prenatal and early postnatal life, when pivotal aspects of human metabolism are established. In this regard, a family history of obesity, body weight and size at birth, and obesity grade during the postnatal life course may play a role in weight-loss ability in adulthood. However, no study has yet investigated the impact of intergenerational obesity and the personal life-course grade of obesity on the ability to lose weight in response to dietary interventions.

Evidence for intergenerational links to obesity has primarily emerged from two-generational studies that have examined the relationship between the body mass index (BMI) of parents in adulthood [5,6,7,8,9,10,11,12], and less frequently at earlier life periods [13], with the offspring’s susceptibility to obesity over a lifetime. The grade of obesity of both parents may influence the offspring’s body composition during childhood [10,13] and adulthood [5,7]. Moreover, some studies have found stronger influences on the offspring’s proneness to obesity for mothers than for fathers [8,11], which suggests that intrauterine mechanisms could be involved. However, Freeman et al. [9] showed that having an overweight or obese father, but a mother of healthy weight, increased the probability of the offspring’s obesity in childhood, suggesting that fathers might also be a key influence. In addition, some studies have been carried out, analyzing the information of three generations (i.e., grandparents, parents, and the offspring) [14,15,16,17,18]. Davis et al. [15] showed grandparental obesity to be associated independently from parental obesity with an increased prevalence of overweight individuals among children aged 5 to 19 years, in a US national sample. Moreover, three studies have provided evidence of a stronger transmission, over three generations, of BMI [16,18] and central adiposity [17] in childhood through the maternal line. Furthermore, maternal and paternal nutritional knowledge and attitudes may influence the effect of dietary interventions on their offspring [19,20]. Nevertheless, to our knowledge, there are no studies analyzing the potential impact of the parental line on body-weight loss in response to dietary interventions.

Studies assessing the association between weight and size at birth with the risk of weight gain and obesity later in life have shown inconclusive results [21,22,23,24,25,26,27,28]. Most studies have reported higher weight and size at birth to be associated with higher BMI in childhood, adolescence and adulthood [21,22,23,25]. A J- or U-shaped association between birth weight and BMI later in life has also been reported [27,28], which indicates a higher propensity for obesity in infants with both low and high birth weights, while other studies have found no association between birth weight and adult BMI [26]. In addition, the development of obesity during postnatal life, including infancy and childhood, may predict obesity in adolescence and adulthood [29,30], and perhaps the ability to lose weight during obesity treatments.

The main objective of the present study was to examine the potential associations between personal early life-course obesity and the ability to lose body weight in response to a dietary intervention in adulthood. As a secondary aim, we examined the relationships between intergenerational obesity, weight and size at birth, and personal early life-course obesity with adult BMI, in an attempt to replicate previous findings.

## 2. Materials and Methods

### 2.1. Study Population

We studied 4264 subjects from the ONTIME study (Obesity, Nutrigenetics, Timing, Mediterranean study) registered at clinicaltrials.gov as NCT02829619. The participants were patients recruited from five obesity clinics in the city of Murcia, located in the southeast of Spain. They voluntarily consented to be recruited for this study. The age of participants ranged from 18 to 80 years. Subjects were excluded if they had some chronic disease such as type 2 diabetes mellitus, hepatic disease, cancer, renal disease, and the use of drugs to promote weight loss. Participants included in this study signed the informed consent and their data were codified to guarantee anonymity. All procedures were in accordance with the Declaration of Helsinki. This study was approved by the Ethical Committee of the University of Murcia.

### 2.2. Intervention and Weight-Loss Effectiveness

A standardized weight-loss program led by certified nutritionists and based on the Mediterranean diet (Garaulet method©) was followed by all the participants [3,31]. This program consisted of attending a 60-minute therapy session per week in small support groups (*n* = 10 per group) during the weight-loss intervention period, which was followed by a 5-month maintenance period. In general, the weight-loss goal for all the participants was to lose at least 5% of the initial body weight. Nevertheless, depending on the initial body weight, the duration of the intervention changed (mean ± SD, 18 ± 14 weeks). Participants with a normal BMI, but who wanted to lose some weight (to achieve a bodyweight always above BMI > 20 kg/m^2^) while learning how to eat following a Mediterranean diet, were also included in the study. Overall, the attrition rate was 32%. The dietary intake of energy was limited to 1200–1800 kcal per day for women and 1500–2000 kcal per day for men, to induce an approximate loss of 0.5–1 kg per week, in order to achieve a total weight loss of 5% of the initial weight. Body weight was recorded weekly throughout the weight-loss phase of the program. Weight-loss effectiveness was evaluated by the total weight loss (kg) achieved, defined as the difference between the initial and the final bodyweight of the participant during the total period of the intervention. In addition, weight loss was also calculated as a percentage of the initial weight. The weight-loss rate was determined as the grams of body weight lost, as tracked weekly during the weight-loss intervention period. During the intervention, participants completed the barriers to weight loss survey [3], which consisted of 37 questions across six sections: (1) meal recording, weight control and weekly interviews; (2) eating habits; (3) portion size; (4) food and drink choices; (5) way of eating and timing; and (6) other obstacles to weight loss, such as places, stress-related eating, eating while watching television, etc. Questions had three possible responses: “never”, “sometimes”, and “always”. A higher cumulative score generally reflects more barriers to weight loss. Scores were dichotomized to the presence (score ≥ 1) or absence (score < 1) of weight-loss barriers.

### 2.3. Assessment of Initial BMI in Adulthood

The bodyweight of participants was measured at baseline and monitored weekly throughout the program. Participants were weighed at baseline after 12 h of fasting, while barefoot, wearing light clothes and using a digital scale (Tanita Corporation of America, Arlington Heights, Illinois, IL, USA) that measured to the nearest 0.1 kg. The height of participants was also measured at baseline using a Harpenden digital stadiometer (rank: 0.7–2.05) (Holtain Ltd. Crosswell, Crymyh, Pembs, UK). Participants were placed in a relaxed, upright position with their heads positioned in the Frankfurt plane. Both height and weight measurements were collected at the same time of the day for all participants. The initial BMI, based on baseline body weight and height measurements, was calculated as weight in kilograms divided by height in square meters (kg/m^2^).

### 2.4. Information on Intergenerational Obesity Grade, Size at Birth and Early-Life Obesity Grade

As part of the ONTIME study, a medical record was obtained from each participant in which the obesity grade history was collected. Information on the obesity grade of grandparents, parents, and throughout lifetime periods, including infancy (birth–23 months of age), childhood (2–12 years of age), and adolescence (13–18 years of age), was reported by participants before starting the weight-loss intervention, following the guidelines of the “Sociedad Española para el Estudio de la Obesidad” (SEEDO) [32]. Categories of the grade of obesity were defined as underweight, normal weight, overweight, and obese, based on the WHO criteria. Information on birth weight (kg) and size at birth, defined as small (< 2500 g), normal (2500–4000 g), and big (>4000 g), was also reported by participants. Children that were overweight and obese were defined by age and sex-specific cut-off points of BMI, based on international data [33].

### 2.5. Statistical Analysis

Associations between adult BMI, total weight loss and rate of weight loss in response to the intervention, and grandparental and parental obesity grade, birth weight, size at birth, and obesity grade in infancy, childhood and adolescence were assessed using multivariate linear regression models. Results are presented as beta coefficients and their corresponding 95% confidence intervals (CI). For birth-weight analyses, the results represent the change per 1-kilogram increase in birth weight to adulthood BMI, to total weight loss (kg), and to the rate of weight loss (grams per week). For size at birth analyses, the reference category was medium size. For the analyses of grandparental, parental, infancy, childhood and adolescence obesity grades, the reference category was normal weight. To study weight loss evolution during treatment, a repeated-measures analysis of variance (ANCOVA) was performed. Final models for BMI in adulthood were adjusted for sex and age; and models for total weight loss were further adjusted for the nutritional clinic, year of assessment, and initial body weight before the dietary intervention. Further adjustment for total energy intake and physical activity level during the intervention did not change the estimates. Nevertheless, when controlling for the main barriers of weight loss, estimates for the association between infant obesity and lower total weight loss in response to the intervention were similar although attenuated (beta coefficient −1.99 kg; 95% CI −4.24, 0.27; *p* = 0.084; data not shown). Finally, analyses were stratified by sex and adult BMI categories. All statistical analyses were performed using the STATA 15 statistical software (Stata Corporation, College Station, TX, USA) and SPSS v20.0 software (IBM Corp., Armonk, NY, USA).

### 2.6. Mediation Analysis

We tested the role of weight and size at birth and overweight–obesity grade in infancy, childhood, and adolescence in mediating the association between maternal and paternal overweight–obesity grade and BMI of the offspring in adulthood. We performed model-based causal mediation analysis using R-package “mediation” [34]. We generated the estimates for the total effect, average direct effect, and average causal mediation effect using a quasi-Bayesian Monte Carlo method based on normal approximation with 2000 simulations, with robust standard errors. The proportion that the mediating variable explains of the association between maternal and paternal overweight–obesity grade and BMI in adulthood was calculated as previously described [35].

## 3. Results

The study population included 4264 adults aged 18 to 80 years (Table 1). The age of participants was 41.5 (±12.9) years (mean (±SD)), and 79% of them were female. The average of total achieved weight loss in response to the intervention was 6.9 (±5.8) kg for the total population; 6.4 (±5.8) kg for women and 9.0 (±6.9) kg for men. The initial BMI of participants was 31.3 (±5.6). Overall, 36% of participants were overweight and 53% were obese. The prevalence of overweight and obesity among women was 40.3% and 46.7%, respectively, and 20.4% and 77.8% among men, respectively. Participants reported a higher prevalence of overweight and obesity among their grandmothers compared to their grandfathers, as well as among their mothers compared to their fathers (32.7% vs. 26.1% for overweight and 16.7% vs. 15.3% for obese, for mother and father, respectively). In the studied population, the reported mean birthweight was 3.5 (±0.6) kg, and 36% and 15% reported being of small and large size at birth, respectively. The prevalence of overweight and obesity steadily increased from infancy to adolescence, varying from 17% to 36.8% for overweight and from 1.3% to 8.2% for obesity, which represent a 2.2-fold change for overweight and a 6.3-fold change for obesity (Table 1).

### 3.1. Total Weight Loss and Weight Loss Evolution in Response to the Intervention

**Figure 1:**
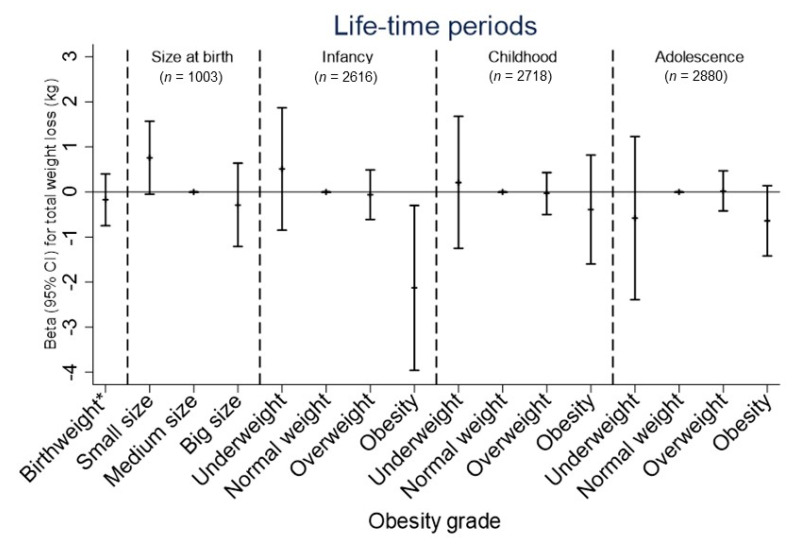
Associations between birth weight, size at birth, and personal life-course obesity grade with total weight loss (kg) in response to the intervention. Lines represent beta coefficient and 95% CI derived from linear regression models. All models adjusted for sex, age, nutritional clinic, year of assessment, and initial body weight. * For birth weight, the coefficient represents the change in total weight loss (kg) as continuous per 1-kg increase in birthweight. For size at birth, a medium size represents the reference category. For infancy, childhood and adolescence periods, a normal weight represents the reference category; *p*-value for infancy obesity grade = 0.023.

Both total weight loss and weight loss rate during the obesity treatment were associated with size at birth and obesity grade during infancy. Participants who reported being small-sized at birth tended to show greater weight loss compared to those who were normal-sized (beta coefficient 0.76 kg; 95% CI, −0.05 to 1.57; *p* = 0.066), although it did not reach statistical significance (Figure 1). Moreover, those who were obese in infancy, but not in childhood or adolescence, showed significant lower total weight loss in response to the intervention (beta coefficient −2.13 kg; 95% CI, −3.96 to −0.30; *p* = 0.023) (Figure 1). After stratifying by adult BMI categories, the association was observed among those who were obese during adulthood (Supplementary Table S1). Results were essentially the same when using total weight loss as a percentage (Supplementary Table S2).

**Figure 2:**
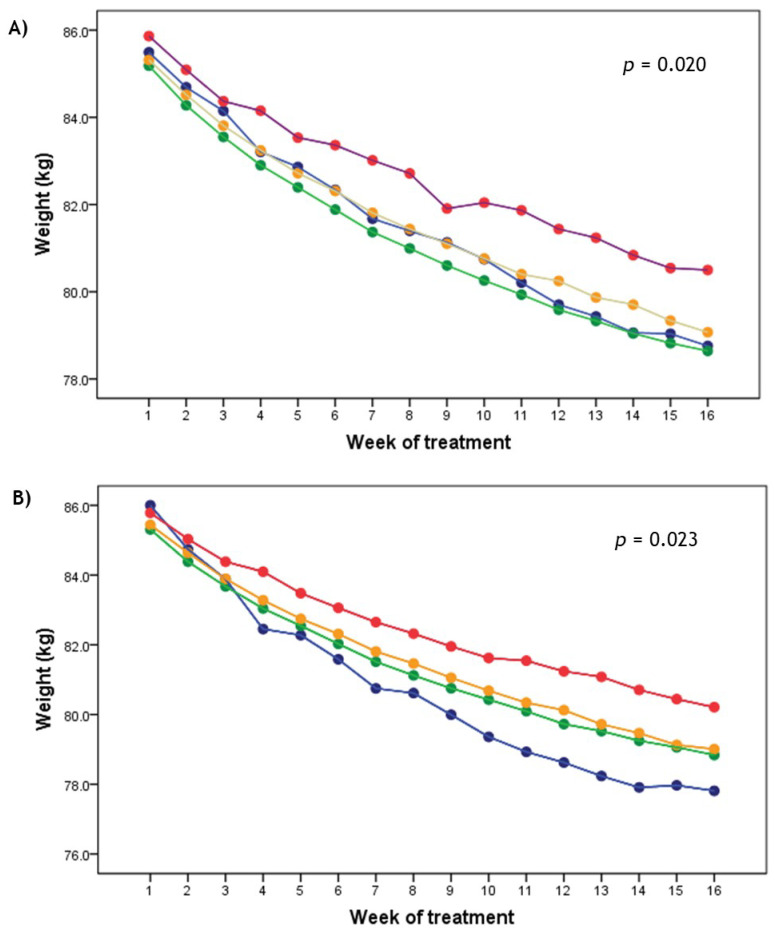
Total weight loss evolution by categories of obesity grade in infancy (**A**) and childhood (**B**) during treatment. A repeated-measures analysis of variance (ANCOVA) was performed. Analyses were adjusted for sex, age, nutritional clinic, year of assessment, and initial body weight. Blue line = underweight; green line = normal weight; yellow line = overweight; red line = obesity.

As for the weight-loss rate and evolution during treatment, participants who were obese in infancy and in childhood displayed a slower rate of weight loss starting from the first week, a difference that gradually increased after the third week of the intervention program (*p* < 0.05; Figure 2). Associations between obesity grade in infancy, childhood and adolescence with total weight loss were in the same direction after stratifying by sex (Supplementary Table S3). Although stronger associations were found in men compared to women, differences did not reach statistical significance (Supplementary Table S3).

An underweight paternal grandfather was associated with higher total weight loss achieved at the end of the intervention (beta coefficient 1.62; 95% CI 0.46 to 2.77; *p* = 0.006), and the paternal grandfather’s overweight status was associated with lower total weight loss (beta coefficient −0.67; 95% CI −1.24 to −0.09; *p* = 0.023) (Table 2). These associations were stronger among those participants with obesity during adulthood (Supplementary Table S4). No associations were found between the parental (mother or father) obesity grade and total weight loss.

### 3.2. Initial BMI in Adulthood

The influence of intergenerational obesity grade on BMI in adulthood before the intervention is shown in Supplementary Table S5. Grandparental overweight and obese statuses were associated with a higher BMI in adulthood (Supplementary Table S5). Moreover, participants with overweight and obese parents had higher BMI in adulthood compared to those with normal-weight parents (Supplementary Table S5). Paternal underweight status tended to be associated with the higher BMI of their offspring in adulthood (beta coefficient 0.84; 95% CI, −0.05 to 1.73; *p* = 0.063). After mutual adjustment, the maternal and paternal obesity grade remained independently associated with higher BMI in adulthood (Supplementary Table S6). After adjustment for the maternal obesity grade, the association between an underweight father and higher BMI in his offspring in adulthood was stronger (beta coefficient 1.44; 95% CI, 0.46 to 2.42; *p* = 0.004) (Supplementary Table S6). Stratified analysis by sex showed that estimates were in the same direction; however, stronger associations with the mothers’ obesity in women and with the fathers’ obesity in men were observed (Supplementary Table S7).

Participants who reported higher weight at birth tended to show a higher BMI in adulthood (BMI estimate for a 1-kilogram increase in birth weight was 0.58; 95% CI, −0.01 to 1.16; *p* = 0.055) (Supplementary Figure S1), although a non-linear relationship was found (Supplementary Figure S2). Accordingly, a U-shaped relationship between the size at birth categories and BMI in adulthood was found. A higher BMI in adulthood was associated with both small size (beta coefficient 0.92; 95% CI, 0.29 to 1.55; *p* = 0.004) and big size (beta coefficient 0.90; 95% CI, 0.04 to 1.76; *p* = 0.041) at birth (Supplementary Figure S1). Underweight status in infancy, childhood, and adolescence was associated with lower BMI in adulthood; however, excess weight and obesity in infancy, childhood, and adolescence were associated with higher BMI in adulthood (Supplementary Figure S1). Associations were in the same direction after stratifying by sex, with stronger associations between infancy and childhood obesity and BMI in adulthood among women (Supplementary Table S8).

### 3.3. Mediation Analysis for BMI in Adulthood

**Figure 3:**
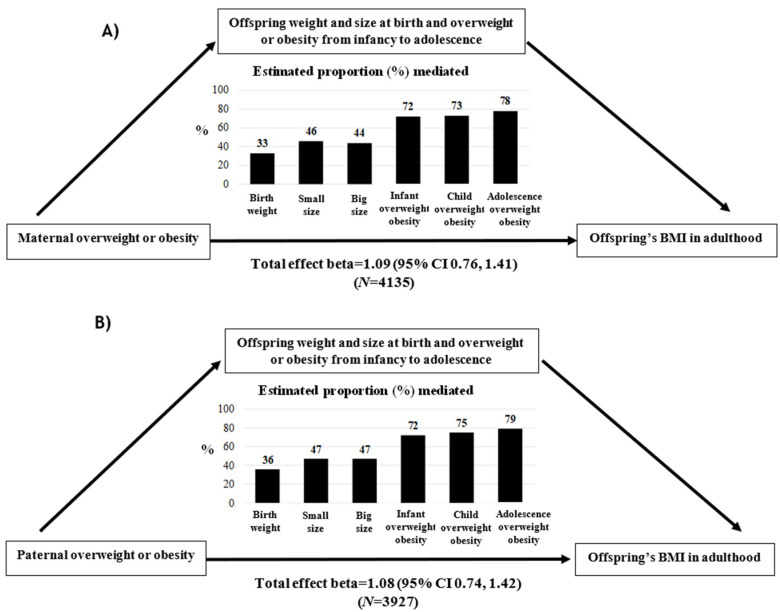
Estimated mediated proportion effect of (**A**) maternal and (**B**) paternal overweight–obesity grade on the offspring’s BMI in adulthood via birth weight, size at birth, and overweight–obesity grade from infancy to adolescence in the ONTIME study.

Further mediation analyses showed evidence for the mediating effects of birth weight, size at birth, and overweight–obesity grade from infancy to adolescence in the association between the maternal (Supplementary Table S9) and paternal (Supplementary Table S10) overweight–obesity grade and offspring’s BMI in adulthood. The estimated mediated proportions gradually increased throughout the life-course periods, with the lowest proportion found for birth weight (33%) and the highest for overweight–obesity in adolescence (79%) (Figure 3).

## 4. Discussion

The present study is the first that investigates the ability to lose weight in response to dietary intervention in relation to intergenerational obesity, body weight and size at birth, and postnatal life-course obesity grade. We show that obesity in infancy and in childhood was associated with lower weight loss and effectiveness of the dietary intervention during adulthood. Those individuals who were obese in infancy lost less body weight than those who were normal weight. As for the weight loss rate and evolution during treatment, participants who were obese in infancy and also in childhood displayed a slower rate of weight loss starting from the first week, a difference that gradually increased after the third week of the intervention program.

### 4.1. Comparisons with Previous Studies

To our knowledge, this is the first study to investigate the potential role of intergenerational obesity, body weight and size at birth, and personal early life-course obesity in an individual’s ability to lose weight during an obesity treatment. We did not find evidence of an association between the parental (both mother and father) obesity grade and the ability to lose weight in response to dietary intervention in adulthood, while participants who reported to be small-sized at birth tended to show higher weight loss compared to those normal-sized. More importantly, a poorer response to the dietary treatment with lower total weight loss (kg) was found among subjects who were obese during infancy, and those subjects who were obese during infancy and also during childhood showed a decreased weekly weight-loss rate and a poorer weight-loss evolution during the treatment. The success of a weight-loss dietary intervention was not related to the degree of obesity during other life-course periods. Some studies have suggested that the first few weeks of life up to the first 2 years (infancy) may be a crucial period for obesity risk later in life [36,37]. Feeding with formula milk rich in macronutrients may be involved in rapid weight gain during the first weeks of life and the increase in adult overweight status [37].

Furthermore, early childhood (between 2 and 6 years of age), a period that encompasses the adiposity rebound, is considered a critical age for the development of sustained obesity [29]. Geserick et al. estimated that the probability that obese children return to a normal weight in adolescence was less than 20% and decreased further with age [29]. It is likely that derived lasting alterations in adipose tissue function and changes in metabolism during infancy and childhood may influence the ability to lose weight in response to treatment later in life. Further studies are warranted to understand the potential mechanisms involved in the association between infancy and childhood obesity, and subsequent difficulties in losing body weight in response to dietary interventions.

The available studies have examined the impact of obesity over three generations by evaluating the offspring’s BMI [16,18] and central adiposity [17] in childhood, and the risk of excess weight (BMI > 95th percentile) up to adolescence [15]. Our study shows an impact of the grandparental obesity grade on the offspring’s BMI beyond adolescence, which supports the transgenerational transmission of the obesity cycle up to three generations and its maintenance in adulthood. We found grandparental overweight and obese status to be associated with higher adult BMI in the offspring, which supports the hypothesis that obesity is in part programmed before conception. In addition, participants with underweight paternal grandmothers, as well as those with underweight fathers but not mothers, had an increased BMI in adulthood. These findings suggest that being underweight through the paternal line may play a role in programming offspring BMI in adulthood.

In our study, we found an association between a paternal grandfather being underweight and higher total weight loss. Moreover, the paternal grandfather’s overweight status was associated with lower total weight loss. It is possible that the grandparents’ behaviors related to their own obesity may have influenced their grandchildren through the children’s parents. Previous two-generational studies have reported both parents’ BMI in adulthood to be positively associated with the offspring’s BMI in adulthood [5,7]. Supporting this, our results showed that both the maternal and paternal obesity grades in adulthood were independently associated with an increase in the offspring’s BMI in adulthood.

Although some previous studies have suggested that one parent, particularly the mother, could have stronger influences on the offspring’s obesity proneness [8,11], we did not detect differences in the associations between the mother’s and father’s obesity and the offspring’s BMI in adulthood in the patients’ studies.

Although mediation analyses did not show any association with weight loss in response to the dietary intervention, we found that associations between parental overweight–obesity status and the offspring’s BMI in adulthood were increasingly explained by weight and size at birth (values ranging from 33% to 47%) and by overweight–obesity status from infancy to adolescence (values ranging from 72% to 79%). In this regard, it is reasonable that older study participants will have had longer exposure to obesogenic environmental factors as shared with their parents, and therefore will show stronger associations. These results suggest that there is a long period during the offspring’s growth to implement obesity prevention strategies.

Mounting evidence in developmental programming, life-course and cross-generation transmission of obesity has increased since Barker proposed the theory of the fetal origins of adult disease [38]. The underlying mechanisms are still unclear, but most probably involve prenatal programming, genetic predisposition and shared environmental factors [39]. It is likely that these associations are largely attributable to their shared environment because many risk factors for obesity, including social environment [40], dietary intake, physical activity patterns and other lifestyle factors, often co-occur within families [41,42]. The lack of an association between weight-loss effectiveness during adulthood and the parental obesity grade may be related to the fact that during the treatment they do not share the same environment. Furthermore, we have shown that one of the main barriers to losing weight is the lack of motivation during the treatment [3], and although this may be highly impacted by the social environment, this impact may be more acute than merely life-course influenced.

However, Cooper et al. showed that parent–offspring BMI associations remained after adjustment for a wide range of lifestyle and socioeconomic factors [7]. Thus, these associations may also reflect genetic and epigenetic factors whose influences are maintained in adult life. The genetic influence on BMI is supported by adoption and twin studies [43,44]. To date, the estimated heritability of BMI ranges from 40% to 70% [45]. Nevertheless, all the genetic variants identified could only explain a relatively small proportion (less than 30%) of the total variation of individual BMI levels and weight variation [46]. While there are association studies demonstrating the possible impact of individual genetic variants on weight-loss effectiveness [47,48,49], novel genome-wide analytical studies (GWAS) are warranted to unravel the genetics of weight loss.

Birth weight as a risk factor for obesity at later ages has been extensively examined with varying results [50]. Accordingly, our results indicate that unfavorable in utero development resulting in a small or big size at birth leads to higher BMI in adulthood. Our findings are in line with previous studies that have shown a U-shaped relationship between size at birth and BMI in adulthood [27,28].

### 4.2. Strengths and Limitations

Among the strengths of this study are the availability of data from three generations and its prospective longitudinal design to assess weight loss effectiveness of dietary intervention in a large sample of subjects. Secondly, weight loss during the follow-up was based on weekly repeated measurements for at least 16 weeks for every participant. Nevertheless, this study has also limitations. The size of compared groups varies, which could influence our results. However, based on the infancy data that shows a significant difference in body weight loss of 1.18 (SD ± 6.08) kg between two groups (overweight *versus* normal weight), it would be necessary to have a sample of at least 953 subjects to achieve a statistical power of 85%. Therefore, we consider that the current study has enough power in every assessed lifetime period to detect a difference in total weight loss of ~1 kg. Information on intergenerational obesity grade, weight and size at birth, and postnatal obesity grade were reported by participants, which may lead to an underreporting bias, particularly in women [51]. This could result in underestimation of the strength of the associations found, since it is likely that the actual obesity grade of mothers and grandmothers was higher. We were not able to control for the participants’ socioeconomic status and lifestyle factors, which could result in residual confounding. Diverse types of barriers are responsible for high interindividual variation in weight loss in response to treatments, which could influence our results. However, although estimates were attenuated, further adjustment for a barrier score developed in our study showed similar results for the association between infancy obesity and lower total weight loss in response to the intervention. Finally, there was some attrition of the obesity program from baseline to the later stages, due to the long-term follow-up to assess weight-loss effectiveness. Therefore, the data of body weight evolution during the dietary treatment may not be representative of the total population.

## 5. Conclusions

The present study suggests that obesity in infancy and childhood may impair weight-loss response to dietary treatments in adulthood. Tackling obesity in early life may improve the effectiveness of weight loss treatments for obesity in adulthood.

## Figures and Tables

**Table 1:** Characteristics of the study population.

	*N*	Mean (SD) or %
Age (years), mean (SD)	4264	41.5 (12.9)
Sex (%)	4264	
Male	895	21.0
Female	3369	79.0
Total weight loss (kg), mean (SD)	3896	6.9 (5.8)
Women	3090	6.4 (5.4)
Men	806	9.0 (6.9)
Adulthood BMI (kg/m^2^), mean (SD)	4264	31.3 (5.6)
<25	453	10.6
25–29.99	1542	36.2
≥30	2269	53.2
Maternal grandmother obesity grade (%)	3607	
Underweight	137	3.8
Normal weight	2265	62.8
Overweight	744	20.6
Obese	461	12.8
Maternal grandfather obesity grade (%)	3571	
Underweight	174	4.9
Normal weight	2694	75.4
Overweight	519	14.5
Obese	187	5.2
Paternal grandmother obesity grade (%)	3530	
Underweight	127	3.6
Normal weight	2243	63.5
Overweight	708	20.1
Obese	452	12.8
Paternal grandfather obesity grade (%)	3420	
Underweight	192	5.6
Normal weight	2562	74.9
Overweight	450	13.2
Obese	216	6.3
Mother obesity grade (%)	4135	
Underweight	130	3.1
Normal weight	1966	47.5
Overweight	1350	32.7
Obese	689	16.7
Father obesity grade (%)	3927	
Underweight	146	3.7
Normal weight	2156	54.9
Overweight	1023	26.1
Obese	602	15.3
Size at birth (%)	1220	
Small	440	36.1
Medium	601	49.2
Big	179	14.7
Birthweight (kg), mean (SD)	753	3.49 (0.65)
Infancy obesity grade (%)	2967	
Underweight	71	2.4
Normal weight	2345	79.0
Overweight	513	17.3
Obese	38	1.3
Childhood obesity grade (%)	3077	
Underweight	62	2.0
Normal weight	2049	66.6
Overweight	874	28.4
Obese	92	3.0
Adolescence obesity grade (%)	3246	
Underweight	38	1.2
Normal weight	1748	53.9
Overweight	1194	36.8
Obese	266	8.2

SD: standard deviation.

**Table 2:** Adjusted associations between intergenerational obesity grade and total weight loss (kilograms) in adulthood, in response to a dietary intervention.

	*N*	Coef. (95% CI)	*p* Value
Maternal grandmother obesity grade	3239		
Underweight	136	0.25 (−1.14, 1.64)	0.726
Normal weight	2001	Ref.	
Overweight	669	−0.15 (−0.63, 0.33)	0.546
Obese	433	0.19 (−0.37, 0.76)	0.504
Maternal grandfather obesity grade	3197		
Underweight	172	0.10 (−1.25, 1.45)	0.882
Normal weight	2393	Ref.	
Overweight	460	−0.49 (−1.03, 0.06)	0.080
Obese	172	0.18 (−0.67, 1.02)	0.682
Paternal grandmother obesity grade	3159		
Underweight	124	0.33 (−0.97, 1.63)	0.622
Normal weight	1979	Ref.	
Overweight	636	0.09 (−0.41, 0.58)	0.727
Obese	420	0.49 (−0.09, 1.07)	0.099
Paternal grandfather obesity grade	3054		
Underweight	190	1.62 (0.46, 2.77)	0.006
Normal weight	2254	Ref.	
Overweight	410	−0.67 (−1.24, −0.09)	0.023
Obese	200	0.28 (−0.51, 1.06)	0.490
Mother obesity grade	3722		
Underweight	130	0.29 (−0.87, 1.44)	0.625
Normal weight	1748	Ref.	
Overweight	1210	−0.04 (−0.43, 0.36)	0.850
Obese	634	−0.12 (−0.62, 0.38)	0.641
Father obesity grade	3514		
Underweight	142	−0.68 (−1.80, 0.45)	0.237
Normal weight	1898	Ref.	
Overweight	918	0.05 (−0.38, 0.48)	0.811
Obese	556	0.08 (−0.44, 0.60)	0.757

Coefficient and 95% confidence intervals derived from linear regression models adjusted for sex, age, nutritional clinic, year of assessment, and initial body weight. For all comparisons, a normal weight represents the reference category.

## Data Availability

The data presented in this study are available on request from the corresponding author.

## Supplementary Materials

The following are available online at https://www.mdpi.com/article/10.3390/nu13072132/s1. Table S1. Associations between birthweight, size at birth, and personal life-course obesity grade with total weight loss (kg) in adulthood stratified by adult body mass index (BMI) categories. Table S2. Associations between intergenerational obesity grade, birthweight, size at birth, and personal life-course obesity grade with total weight loss expressed as percentage in response to the intervention. Table S3. Associations between birthweight, size at birth, and personal life-course obesity grade with total weight loss (kg) in adulthood stratified by sex. Table S4. Adjusted associations between intergenerational obesity grade and total weight loss (kg) in adulthood stratified by adult body mass index (BMI) categories. Table S5. Adjusted associations between intergenerational obesity grade and body mass index (BMI) in adulthood. Table S6. Mutually-adjusted associations between grandparental and parental obesity grade with body mass index (BMI) in adulthood. Table S7. Adjusted associations between intergenerational obesity grade and body mass index (BMI) in adulthood stratified by sex. Figure S1. Associations between birthweight, size at birth, and personal life-course obesity grade with adult body mass index (BMI) in response to the intervention. Figure S2. The relation (and 95% confidence levels) of birth weight with body mass index (BMI) in adulthood. General additive models adjusted for sex and age. Table S8. Associations between birthweight, size at birth, and personal life-course obesity grade with body mass index (BMI) in adulthood stratified by sex. Table S9. Mediation analysis examining the mediated effect of maternal overweight-obesity on BMI in adulthood in the ONTIME study. Table S10. Mediation analysis examining the mediated effect of paternal overweight-obesity on BMI in adulthood in the ONTIME study.

## References

[1] GBD 2017 Risk Factor Collaborators (2018). Global, regional, and national comparative risk assessment of 84 behavioural, environmental and occupational, and metabolic risks or clusters of risks for 195 countries and territories, 1990–2017: A systematic analysis for the Global Burden of Disease Study 2017. Lancet.

[2] Ashton L.M., Sharkey T., Whatnall M.C., Haslam R.L., Bezzina A., Aguiar E.J., Collins C.E., Hutchesson M.J. (2020). Which behaviour change techniques within interventions to prevent weight gain and/or initiate weight loss improve adiposity outcomes in young adults? A systematic review and meta-analysis of randomized controlled trials. Obes. Rev..

[3] Corbalán M.D., Morales E.M., Canteras M., Espallardo A., Hernández T., Garaulet M. (2009). Effectiveness of cognitive-behavioral therapy based on the Mediterranean diet for the treatment of obesity. Nutrition.

[4] Hainer V., Zamrazilová H., Spálová J., Hainerová I., Kunesová M., Aldhoon B., Bendlová B. (2008). Role of hereditary factors in weight loss and its maintenance. Physiol. Res..

[5] Whitaker R.C., Wright J.A., Pepe M.S., Seidel K.D., Dietz W.H. (1997). Predicting obesity in young adulthood from childhood and parental obesity. N. Engl. J. Med..

[6] Gordon-Larsen P., Adair L.S., Suchindran C.M. (2007). Maternal obesity is associated with younger age at obesity onset in U.S. adolescent offspring followed into adulthood. Obesity.

[7] Cooper R., Hyppönen E., Berry D., Power C. (2010). Associations between parental and offspring adiposity up to midlife: The contribution of adult lifestyle factors in the 1958 British Birth Cohort Study. Am. J. Clin. Nutr..

[8] Whitaker K.L., Jarvis M.J., Beeken R.J., Boniface D., Wardle J. (2010). Comparing maternal and paternal intergenerational transmission of obesity risk in a large population-based sample. Am. J. Clin. Nutr..

[9] Freeman E., Fletcher R., Collins C.E., Morgan P.J., Burrows T., Callister R. (2012). Preventing and treating childhood obesity: Time to target fathers. Int. J. Obes..

[10] Birbilis M., Moschonis G., Mougios V., Manios Y. (2013). Obesity in adolescence is associated with perinatal risk factors, parental BMI and sociodemographic characteristics. Eur. J. Clin. Nutr..

[11] Linabery A.M., Nahhas R.W., Johnson W., Choh A.C., Towne B., Odegaard A.O., Czerwinski S.A., Demerath E.W. (2013). Stronger influence of maternal than paternal obesity on infant and early childhood body mass index: The Fels Longitudinal Study. Pediatric Obes..

[12] Chaparro M.P., Koupil I., Byberg L. (2017). Maternal pre-pregnancy BMI and offspring body composition in young adulthood: The modifying role of offspring sex and birth order. Public Health Nutr..

[13] Li L., Law C., Lo Conte R., Power C. (2009). Intergenerational influences on childhood body mass index: The effect of parental body mass index trajectories. Am. J. Clin. Nutr..

[14] Guillaume M., Lapidus L., Beckers F., Lambert A., Björntorp P. (1995). Familial trends of obesity through three generations: The Belgian-Luxembourg child study. J. Obes. Relat. Metab. Disord..

[15] Davis M.M., McGonagle K., Schoeni R.F., Stafford F. (2008). Grandparental and parental obesity influences on childhood overweight: Implications for primary care practice. J. Am. Board Fam. Med..

[16] Murrin C.M., Kelly G.E., Tremblay R.E., Kelleher C.C. (2012). Body mass index and height over three generations: Evidence from the Lifeways cross-generational cohort study. BMC Public Health.

[17] Somerville R., Khalil H., Segurado R., Mehegan J., Viljoen K., Heinen M., Murrin C., Kelleher C.C. (2018). Childhood central adiposity at ages 5 and 9 shows consistent relationship with that of the maternal grandmother but not other grandparents. Pediatric Obes..

[18] McKey S., Heinen M., Mehegan J., Somerville R., Khalil H., Segurado R., Murrin C., Kelleher C.C. (2017). Lifeways Cross-Generation Cohort Study Steering Group. Predictors of adults’ body mass index and the association with index child’s infant birth weight, in the Lifeways Cross-Generation Cohort Study of a thousand families in the Republic of Ireland. J. Dev. Orig. Health Dis..

[19] Al-Shookri A., Al-Shukaily L., Hassan F., Al-Sheraji S., Al-Tobi S. (2011). Effect of Mothers Nutritional Knowledge and Attitudes on Omani Children’s Dietary Intake. Oman Med. J..

[20] Romanos-Nanclares A., Zazpe I., Santiago S., Marín L., Rico-Campà A., Martín-Calvo N. (2018). Influence of Parental Healthy-Eating Attitudes and Nutritional Knowledge on Nutritional Adequacy and Diet Quality among Preschoolers: The SENDO Project. Nutrients.

[21] Eriksson J., Forsén T., Tuomilehto J., Osmond C., Barker D. (2001). Size at birth, childhood growth and obesity in adult life. Int. J. Obes. Relat. Metab. Disord..

[22] Gunnarsdottir I., Birgisdottir B.E., Benediktsson R., Gudnason V., Thorsdottir I. (2004). Association between size at birth, truncal fat and obesity in adult life and its contribution to blood pressure and coronary heart disease; study in a high birth weight population. Eur. J. Clin. Nutr..

[23] Pilgaard K., Færch K., Poulsen P., Larsen C., Andersson E.A., Pisinger C., Toft U., Aadahl M., Pedersen O., Hansen T. (2010). Impact of size at birth and prematurity on adult anthropometry in 4744 middle-aged Danes—The Inter99 study. J. Dev. Orig. Health Dis..

[24] Evensen E., Emaus N., Kokkvoll A., Wilsgaard T., Furberg A.S., Skeie G. (2017). The relation between birthweight, childhood body mass index, and overweight and obesity in late adolescence: A longitudinal cohort study from Norway, The Tromsø Study, Fit Futures. BMJ Open.

[25] Jelenkovic A., Yokoyama Y., Sund R., Pietiläinen K.H., Hur Y.M., Willemsen G., Bartels M., van Beijsterveldt T.C.E.M., Ooki S., Saudino K.J. (2017). Association between birthweight and later body mass index: An individual-based pooled analysis of 27 twin cohorts participating in the CODATwins project. Int. J. Epidemiol..

[26] Johnsson I.W., Ahlsson F., Gustafsson J. (2019). High birthweight was not associated with altered body composition or impaired glucose tolerance in adulthood. Acta Paediatr..

[27] Gaskins R.B., LaGasse L.L., Liu J., Shankaran S., Lester B.M., Bada H.S., Bauer C.R., Das A., Higgins R.D., Roberts M. (2010). Small for gestational age and higher birth weight predict childhood obesity in preterm infants. Am. J. Perinatol..

[28] Stettler N., Iotova V. (2010). Early growth patterns and long-term obesity risk. Curr. Opin. Clin. Nutr. Metab. Care.

[29] Geserick M., Vogel M., Gausche R., Lipek T., Spielau U., Keller E., Pfäffle R., Kiess W., Körner A. (2018). Acceleration of BMI in Early Childhood and Risk of Sustained Obesity. N. Engl. J. Med..

[30] Ward Z.J., Long M.W., Resch S.C., Giles C.M., Cradock A.L., Gortmaker S.L. (2017). Simulation of Growth Trajectories of Childhood Obesity into Adulthood. N. Engl. J. Med..

[31] Serra-Majem L., Aranceta J. (2001). Nutritional objectives for the Spanish population. Consensus from the Spanish Society of Community Nutrition; SENC Working Group on Nutritional Objectives for the Spanish Population. Spanish Society of Community Nutrition. Public Health Nutr..

[32] (2000). SEEDO’2000 consensus for the evaluation of overweight and obesity and the establishment of criteria for therapeutic intervention; Sociedad Española para el Estudio de la Obesidad. Med. Clin. (Barc.).

[33] Cole T.J., Bellizzi M.C., Flegal K.M., Dietz W.H. (2000). Establishing a standard definition for child overweight and obesity worldwide: International survey. BMJ.

[34] Tingley D., Yamamoto T., Hirose K., Keele L., Imai K. (2014). mediayion: R package for causal mediation analysis. J. Stat. Softw..

[35] Imai K., Keele L., Yamamoto T. (2010). Identification, inference and sensitivity analysis for causal mediation effects. Stat. Sci..

[36] Toschke A.M., Grote V., Koletzko B., von Kries R. (2004). Identifying children at high risk for overweight at school entry by weight gain during the first 2 years. Arch. Pediatrics Adolesc. Med..

[37] Stettler N., Stallings V.A., Troxel A.B., Zhao J., Schinnar R., Nelson S.E., Ziegler E.E., Strom B.L. (2005). Weight gain in the first week of life and overweight in adulthood: A cohort study of European American subjects fed infant formula. Circulation.

[38] Barker D., Osmond C. (1986). Infant mortality, childhood nutrition, and ischaemic heart disease in England and Wales. Lancet.

[39] Segal N.L., Feng R., McGuire S.A., Allison D.B., Miller S. (2009). Genetic and environmental contributions to body mass index: Comparative analysis of monozygotic twins, dizygotic twins and same-age unrelated siblings. Int. J. Obes..

[40] Lee A., Cardel M., Donahoo W.T., Feingold K.R., Anawalt B., Boyce A. (2000). Social and Environmental Factors Influencing Obesity. [Updated 12 October 2019]. Endotext [Internet].

[41] Simonen R.L., Perusse L., Rankinen T., Rice T., Rao D.C., Bouchard C. (2002). Familial aggregation of physical a tivity levels in the Québec Family Study. Med. Sci. Sports Exerc..

[42] Provencher V., Pérusse L., Bouchard L., Drapeau V., Bouchard C., Rice T., Rao D.C., Tremblay A., Després J.P., Lemieux S. (2005). Familial resemblance in eating behaviors in men and women from the Quebec Family Study. Obes. Res..

[43] Stunkard A.J., Sørensen T.I., Hanis C., Teasdale T.W., Chakraborty R., Schull W.J., Schulsinger F. (1986). An adoption study of human obesity. N. Engl. J. Med..

[44] Sørensen T.I., Holst C., Stunkard A.J. (1998). Adoption study of environmental modifications of the genetic influences on obesity. Int. J. Obes. Relat. Metab. Disord..

[45] Bray M.S., Loos R.J., McCaffery J.M., Ling C., Franks P.W., Weinstock G.M., Snyder M.P., Vassy J.L., Agurs-Collins T. (2016). Conference Working Group. NIH working group report-using genomic information to guide weight management: From universal to precision treatment. Obesity.

[46] Elks C.E., den Hoed M., Zhao J.H., Sharp S.J., Wareham N.J., Loos R.J., Ong K.K. (2012). Variability in the heritability of body mass index: A systematic review and meta-regression. Front. Endocrinol. (Lausanne).

[47] Garaulet M., Sánchez-Moreno C., Smith C.E., Lee Y.C., Nicolás F., Ordovás J.M. (2011). Ghrelin, sleep reduction and evening preference: Relationships to CLOCK 3111 T/C SNP and weight loss. PLoS ONE.

[48] Garaulet M., Esteban Tardido A., Lee Y.C., Smith C.E., Parnell L.D., Ordovás J.M. (2012). SIRT1 and CLOCK 3111T > C combined genotype is associated with evening preference and weight loss resistance in a behavioral therapy treatment for obesity. Int. J. Obes..

[49] Garaulet M., Vera B., Bonnet-Rubio G., Gómez-Abellán P., Lee Y.C., Ordovás J.M. (2016). Lunch eating predicts weight-loss effectiveness in carriers of the common allele at PERILIPIN1: The ONTIME (Obesity, Nutrigenetics, Timing, Mediterranean) study. Am. J. Clin. Nutr..

[50] Woo Baidal J.A., Locks L.M., Cheng E.R., Blake-Lamb T.L., Perkins M.E., Taveras E.M. (2016). Risk Factors for Childhood Obesity in the First 1000 Days: A Systematic Review. Am. J. Prev. Med..

[51] Merrill R.M., Richardson J.S. (2009). Validity of self-reported height, weight, and body mass index: Findings from the National Health and Nutrition Examination Survey, 2001–2006. Prev. Chronic. Dis..

