# Supplementary material for: Infancy and Childhood Obesity Grade Predicts Weight Loss in Adulthood: The ONTIME Study
Source: Nutrients. 2021 Jun 22;13(7):2132. doi: 10.3390/nu13072132 (PMC8308354; doi:10.3390/nu13072132)
Supplement: Supplementary file 1 [file nutrients-13-02132-s001.zip › nutrients-1247850-supplementary.pdf]

Online-only Supplement for

Infancy and childhood obesity grade predicts weight loss in adulthood: the ONTIME study

Eva Morales, Nathaly Torres-Castillo, Marta Garaulet,

Table S1. Associations between birthweight, size at birth, and personal life-course obesity grade with total weight loss (kg) in adulthood stratified by adult body mass index (BMI) categories.

Table S2. Associations between intergenerational obesity grade, birthweight, size at birth, and personal life-course obesity grade with total weight loss expressed as percentage in response to the intervention.

Table S3. Associations between birthweight, size at birth, and personal life-course obesity grade with total weight loss (kg) in adulthood stratified by sex.

Table S4. Adjusted associations between intergenerational obesity grade and total weight loss (kg) in adulthood stratified by adult body mass index (BMI) categories.

Table S5. Adjusted associations between intergenerational obesity grade and body mass index (BMI) in adulthood.

Table S6. Mutually-adjusted associations between grandparental and parental obesity grade with body mass index (BMI) in adulthood.

Table S7. Adjusted associations between intergenerational obesity grade and body mass index (BMI) in adulthood stratified by sex.

Figure S1. Associations between birthweight, size at birth, and personal life-course obesity grade with adult body mass index (BMI) in response to the intervention.

Figure S2. The relation (and 95% confidence levels) of birth weight with body mass index (BMI) in adulthood. General additive models adjusted for sex and age.

Table S8. Associations between birthweight, size at birth, and personal life-course obesity grade with body mass index (BMI) in adulthood stratified by sex.

Table S9. Mediation analysis examining the mediated effect of maternal overweight-obesity on BMI in adulthood in the ONTIME study.

Table S10. Mediation analysis examining the mediated effect of paternal overweight-obesity on BMI in adulthood in the ONTIME study.

Table S1. Associations between birthweight, size at birth, and personal life-course obesity grade with total weight loss (kg) in adulthood stratified by adult body mass index (BMI) categories.

	Normal BMI (<25 kg/m²)			Overweight (BMI 25-29.99 kg/m²)			Obesity (BMI ≥30 kg/m²)		
	**N**	**Coef. (95% CI)**	**P value**	**N**	**Coef. (95% CI)**	**P value**	**N**	**Coef. (95% CI)**	**P value**
**Birth weight**	**72**	-0.43 (-1.66, 0.80)	0.483	**211**	-0.16 (-0.99, 0.67)	0.707	**267**	-0.16 (-1.08, 0.77)	0.737
**Size at birth**	**111**			**379**			**513**		
Small	10	-0.40 (-1.57, 0.76)	0.496	47	0.88 (-0.12, 1.89)	0.085	83	0.95 (-0.47, 2.37)	0.190
Medium	65	Ref.		179	Ref.		209	Ref.	
Big	36	-2.18 (-4.01, -0.36)	0.020	153	0.36 (-0.88, 1.60)	0.569	221	-0.47 (-1.96, 1.03)	0.538
**Infancy**	**291**			**973**			**1349**		
Underweight	15	0.32 (-1.19, 1.83)	0.678	20	0.54 (-1.32, 2.39)	0.570	26	0.62 (-1.87, 3.12)	0.624
Normal weight	257	Ref.		802	Ref.		995	Ref.	
Overweight	18	0.81 (-0.60, 2.22)	0.258	144	-0.34 (-1.10, 0.41)	0.372	302	0.11 (-0.73, 0.94)	0.798
Obesity	1	-3.18 (-8.75, 2.39)	0.263	7	0.61 (-2.52, 3.74)	0.703	26	-2.60 (-5.12, -0.08)	0.043
**Childhood**	**294**			**1005**			**1416**		
Underweight	17	0.70 (-0.72, 2.13)	0.333	18	-0.45 (-2.38, 1.48)	0.649	18	0.74 (-2.24, 3.72)	0.625
Normal weight	234	Ref.		709	Ref.		837	Ref.	
Overweight	42	0.55 (-0.43, 1.54)	0.271	256	-1.14 (-1.75, -0.54)	<0.001	500	0.56 (-0.17, 1.28)	0.132
Obesity	1	0.11 (-5.61, 5.83)	0.970	22	-0.28 (-2.09, 1.52)	0.758	61	-0.41 (-2.13, 1.30)	0.636
**Adolescence**	**309**			**1063**			**1505**		
Underweight	10	0.76 (-1.05, 2.58)	0.409	17	-0.42 (-2.39, 1.56)	0.677	7	-2.19 (-6.95, 2.56)	0.366
Normal weight	220	Ref.		614	Ref.		681	Ref.	
Overweight	59	-0.31 (-1.18, 0.55)	0.476	368	-0.74 (-1.29, 0.18)	0.009	649	0.56 (0.17, 1.30)	0.132
Obesity	20	-0.10 (-1.62, 1.42)	0.898	64	-1.27 (-2.40, -0.14)	0.028	168	-0.11 (-1.30, 1.08)	0.857

Coefficient and 95% confidence intervals derived from linear regression models adjusted for age, nutritional clinic, year of assessment, and initial body weight. For comparisons, medium size at birth and normal weight represent the reference categories.

Table S2. Associations between intergenerational obesity grade, birthweight, size at birth, and personal life-course obesity grade with total weight loss expressed as percentage in response to the intervention.

	N	Coef. (95% CI)	P value
**Maternal grandmother obesity**	**3239**		
Underweight	136	0.08 (-1.47, 1.62)	0.923
Normal weight	2001	Ref.	
Overweight	669	-0.03 (-0.56, 0.50)	0.910
Obesity	433	0.23 (-0.40, 0.87)	0.468
**Maternal grandfather obesity**	**3197**		
Underweight	172	0.24 (-1.26, 1.74)	0.752
Normal weight	2393	Ref.	
Overweight	460	-0.44 (-1.04, 1.69)	0.157
Obesity	172	0.17 (-0.76, 1.11)	0.713
**Paternal grandmother obesity**	**3159**		
Underweight	124	0.02 (-1.43, 1.47)	0.97
Normal weight	1979	Ref.	
Overweight	636	0.13 (-0.42, 0.67)	0.649
Obesity	420	0.65 (0.001, 1.29)	0.050
**Paternal grandfather obesity**	**3054**		
Underweight	190	1.77 (0.49, 3.05)	0.007
Normal weight	2254	Ref.	
Overweight	410	-0.74 (-1.38, -0.10)	0.024
Obesity	200	0.32 (-0.55, 1.19)	0.473
**Mother obesity grade**	**3722**		
Underweight	130	0.44 (-0.84, 1.72)	0.501
Normal weight	1748	Ref.	
Overweight	1210	-0.04 (-0.48, 0.39)	0.842
Obesity	634	-0.06 (-0.62, 0.49)	0.821
**Father obesity grade**	**3514**		
Underweight	142	-0.86 (-2.11, 0.39)	0.175
Normal weight	1898	Ref.	
Overweight	918	0.09 (-0.38, 0.57)	0.701
Obesity	556	0.12 (-0.45, 0.69)	0.686
**Size at birth**	**1003**		
Small	140	0.85 (-0.04, 1.75)	0.061
Medium	453	Ref.	
Big	140	-0.19 (-1.21, 0.83)	0.710
**Birthweight**	**550**	-0.17 (-0.83, 0.48)	0.603
**Infancy obesity grade**	**2616**		
Underweight	61	0.22 (-1.32, 1.76)	0.778
Normal weight	2057	Ref.	
Overweight	464	-0.002 (-0.62, 0.62)	0.995
Obesity	34	-2.01 (-4.07, 0.05)	0.057
**Childhood obesity grade**	**2718**		
Underweight	53	-0.04 (-1.69, 1.61)	0.963
Normal weight	1781	Ref.	
Overweight	800	-0.07 (-0.59, 0.45)	0.789
Obesity	84	-0.17 (-1.54, 1.20)	0.812
**Adolescence obesity grade**	**2880**		
Underweight	34	-0.85 (-2.89, 1.19)	0.412
Normal weight	1516	Ref.	
Overweight	1078	-0.02 (-0.52, 0.48)	0.947
Obesity	252	-0.69 (-1.57, 0.19)	0.123

All models adjusted for sex, age, nutritional clinic, year of assessment, and initial body weight. [*]For birthweight, the coefficient represents the change in total weight loss (kg) as continuous per 1 kg increase in birthweight. For size at birth, medium size represents the reference category. For infancy, childhood and adolescence periods, normal weight represents the reference category.

Table S3. Associations between birthweight, size at birth, and personal life-course obesity grade with total weight loss (kg) in adulthood stratified by sex.

	Women			Men		
	**N**	**Coef. (95% CI)**	**P value**	**N**	**Coef. (95% CI)**	**P value**
**Birth weight**	**422**	0.12 (-0.49, 0.72)	0.704	**128**	-0.99 (-2.47, 0.48)	0.184
**Size at birth**	**767**			**236**		
Small	85	0.61 (-0.16, 1.38)	0.121	55	1.63 (-1.10, 4.35)	0.240
Medium	356	Ref.		97	Ref.	
Big	326	0.07 (-0.93, 1.07)	0.897	84	-0.55 (-2.72, 1.61)	0.616
**Infancy**	**2094**			**522**		
Underweight	54	0.20 (-1.17, 1.58)	0.722	7	2.99 (-1.82, 7.80)	0.222
Normal weight	1667	Ref.		390	Ref.	
Overweight	348	-0.03 (-0.62, 0.57)	0.933	116	-0.26 (-1.63, 1.12)	0.715
Obesity	25	-1.80 (-3.82, 0.22)	0.081	9	-3.19 (-7.48, 1.09)	0.144
**Childhood**	**2170**			**548**		
Underweight	45	0.28 (-1.23, 1.80)	0.716	8	-0.29 (-4.75, 4.17)	0.898
Normal weight	1452	Ref.		329	Ref.	
Overweight	610	-0.23 (-0.73, 0.26)	0.356	190	0.52 (-0.64, 1.69)	0.376
Obesity	63	0.20 (-1.13, 1.53)	0.766	21	-2.10 (-4.99, 0.79)	0.154
**Adolescence**	**2313**			**567**		
Underweight	29	-0.62 (-2.49, 1.25)	0.514	5	-0.89 (-6.49, 4.71)	0.755
Normal weight	1211	Ref.		305	Ref.	
Overweight	876	-0.12 (-0.60, 0.35)	0.614	202	0.55 (-0.62, 1.72)	0.357
Obesity	197	-0.29 (-1.14, 0.56)	0.504	55	-1.89 (-3.84, 0.06)	0.057

Coefficient and 95% confidence intervals derived from linear regression models adjusted for age, nutritional clinic, year of assessment, and initial body weight. For comparisons, medium size at birth and normal weight represent the reference categories.

Table S4. Adjusted associations between intergenerational obesity grade and total weight loss (kg) in adulthood stratified by adult body mass index (BMI) categories.
Coefficient and 95% confidence intervals derived from linear regression models adjusted for age, nutritional clinic, year of assessment, and initial body weight. For all comparisons, normal weight represents the reference category

	Normal BMI (<25 kg/m²)			Overweight (BMI 25-29.99 kg/m²)			Obesity (BMI ≥30 kg/m²)		
	**N**	**Coef. (95% CI)**	**P value**	**N**	**Coef. (95% CI)**	**P value**	**N**	**Coef. (95% CI)**	**P value**
**Maternal grandmother obesity grade**	**330**			**1177**			**1731**		
Underweight	12	-0.90 (-3.76, 1.96)	0.535	54	0.48 (-1.28, 2.25)	0.591	69	0.76 (-1.45, 2.97)	0.501
Normal weight	205	Ref.		740	Ref.		1056	Ref.	
Overweight	68	0.84 (0.05, 1.63)	0.036	251	0.02 (-0.56, 0.61)	0.937	350	-0.45 (-1.24, 0.34)	0.265
Obesity	45	0.21 (-0.73, 1.15)	0.660	132	0.12 (-0.64, 0.89)	0.747	256	0.17 (-0.72, 1.07)	0.701
**Maternal grandfather obesity grade**	**331**			**1169**			**1696**		
Underweight	17	0.64 (-1.69, 2.97)	0.591	68	0.82 (-0.72, 2.37)	0.298	86	-0.36 (-2.73, 2.00)	0.764
Normal weight	264	Ref.		880	Ref.		1249	Ref.	
Overweight	36	0.44 (-0.58, 1.45)	0.399	162	-0.37 (-1.07, 0.32)	0.290	262	-0.71 (-1.58, 0.16)	0.111
Obesity	14	-1.47 (-3.01, 0.08)	0.062	59	0.41 (-0.67, 1.50)	0.453	99	0.33 (-1.00, 1.67)	0.624
**Paternal grandmother obesity grade**	**327**			**1150**			**1680**		
Underweight	13	0.34 (-1.75, 2.42)	0.749	45	0.22 (-1.36, 1.80)	0.784	66	0.74 (-1.46, 2.94)	0.510
Normal weight	235	Ref.		731	Ref.		1011	Ref.	
Overweight	52	0.66 (-0.19, 1.52)	0.129	244	0.14 (-0.46, 0.73)	0.652	340	-0.11 (-0.92, 0.71)	0.799
Obesity	27	1.04 (-0.09, 2.17)	0.071	130	0.68 (-0.08, 1.45)	0.081	263	0.30 (-0.60, 1.20)	0.512
**Paternal grandfather obesity grade**	**310**			**1109**			**1633**		
Underweight	14	0.73 (-1.14, 2.60)	0.442	77	1.12 (-0.24, 2.48)	0.106	98	2.06 (0.08, 4.04)	0.041
Normal weight	251	Ref.		812	Ref.		1190	Ref.	
Overweight	31	-0.40 (-1.48, 0.68)	0.469	156	-0.31 (-1.00, 0.39)	0.385	223	-1.04 (-1.98, -0.10)	0.030
Obesity	14	-0.49 (-2.04, 1.06)	0.531	64	0.21 (-0.82, 1.25)	0.682	122	0.34 (-0.87, 1.56)	0.577
**Mother obesity grade**	**379**			**1353**			**1987**		
Underweight	18	0.16 (-1.43, 1.76)	0.840	58	1.11 (-0.30, 2.52)	0.123	52	0.42 (-1.66, 2.51)	0.692
Normal weight	212	Ref.		680	Ref.		855	Ref.	
Overweight	115	0.63 (-0.01, 1.28)	0.054	430	0.07 (-0.42, 0.55)	0.789	665	-0.18 (-0.84, 0.48)	0.598
Obesity	34	-0.04 (-1.07, 0.99)	0.935	185	-0.04 (-0.70, 0.61)	0.899	415	-0.16 (-0.93, 0.60)	0.674
**Father obesity grade**	**360**			**1269**			**1884**		
Underweight	9	-0.67 (-2.83, 1.49)	0.543	65	-0.37 (-1.67, 0.92)	0.570	68	-0.53 (-2.44, 1.37)	0.583
Normal weight	230	Ref.		712	Ref.		955	Ref.	
Overweight	81	0.20 (-0.51, 0.91)	0.573	325	0.34 (-0.19, 0.87)	0.214	512	-0.32 (-1.02, 0.38)	0.370
Obesity	40	0.67 (-0.25, 1.59)	0.152	167	0.02 (-0.66, 0.70)	0.950	349	-0.01 (-0.82, 0.80)	0.981

Table S5. Adjusted associations between intergenerational obesity grade and body mass index (BMI) in adulthood.

	N	Coef. (95% CI)	P value
**Maternal grandmother obesity grade**	**3607**		
Underweight	137	0.52 (-0.39, 1.44)	0.266
Normal weight	2265	Ref.	
Overweight	744	0.15 (-0.29, 0.59)	0.506
Obesity	461	1.21 (0.69, 1.75)	<0.001
**Maternal grandfather obesity grade**	**3571**		
Underweight	174	0.09 (-0.72, 0.91)	0.823
Normal weight	2694	Ref.	
Overweight	519	0.59 (0.09, 1.10)	0.021
Obesity	184	1.85 (1.05, 2.65)	<0.001
**Paternal grandmother obesity grade**	**3530**		
Underweight	127	1.06 (0.11, 2.01)	0.029
Normal weight	2243	Ref.	
Overweight	708	0.63 (0.18, 1.08)	0.006
Obesity	452	1.68 (1.14, 2.22)	<0.001
**Paternal grandfather obesity grade**	**3420**		
Underweight	192	0.51 (-0.27, 1.30)	0.199
Normal weight	2562	Ref.	
Overweight	450	0.41 (-0.12, 0.95)	0.130
Obesity	216	1.40 (0.65, 2.14)	<0.001
**Mother obesity grade**	**4135**		
Underweight	130	-0.44 (-1.37, 0.50)	0.359
Normal weight	1966	Ref.	
Overweight	1350	0.60 (0.23, 0.96)	0.001
Obesity	689	1.98 (1.52, 2.44)	<0.001
**Father obesity grade**	**3927**		
Underweight	146	0.84 (-0.05, 1.73)	0.063
Normal weight	2156	Ref.	
Overweight	1023	0.65 (0.26, 1.05)	0.001
Obesity	602	1.95 (1.47, 2.43)	<0.001

Coefficient and 95% confidence intervals derived from linear regression models adjusted for sex and age. For all comparisons, normal weight represents the reference category.

Table S6. Mutually-adjusted associations between grandparental and parental obesity grade with body mass index (BMI) in adulthood.

	N	Coef. (95% CI)	P value
**Maternal grandparental obesity grade**	**3499**		
**Maternal grandmother**			
Underweight		0.88 (-0.45, 2.20)	0.194
Normal weight		Ref.	
Overweight		0.02 (-0.46, 0.49)	0.950
Obesity		0.82 (0.26, 1.38)	0.004
**Maternal grandfather**			
Underweight		-0.31 (-1.49, 0.88)	0.612
Normal weight		Ref.	
Overweight		0.59 (0.06, 1.13)	0.030
Obesity		1.66 (0.83, 2.50)	<0.001
**Paternal grandparental obesity grade**	**3333**	**Coef. (95% CI)**	
**Paternal grandmother**			
Underweight		1.20 (-0.06, 2.46)	0.063
Normal weight		Ref.	
Overweight		0.61 (0.12, 1.10)	0.015
Obesity		1.44 (0.86, 2.03)	<0.001
**Paternal grandfather**			
Underweight		-0.05 (-1.08, 0.97)	0.920
Normal weight		Ref.	
Overweight		0.28 (-0.30, 0.86)	0.342
Obesity		0.93 (0.14, 1.71)	0.022
**Parental obesity grade**	**3919**	**Coef. (95% CI)**	
**Mother obesity grade**			
Underweight		-0.81 (-1.86, 0.25)	0.133
Normal weight		Ref.	
Overweight		0.58 (0.20, 0.96)	0.003
Obesity		1.74 (1.26, 2.22)	<0.001
**Father obesity grade**			
Underweight		1.44 (0.46, 2.42)	0.004
Normal weight		Ref.	
Overweight		0.58 (0.18, 0.98)	0.005
Obesity		1.54 (1.05, 2.03)	<0.001

Coefficient and 95% confidence intervals derived from linear regression models adjusted for sex and age. For all comparisons, normal weight represents the reference category.

Table S7. Adjusted associations between intergenerational obesity grade and body mass index (BMI) in adulthood stratified by sex.

	Women			Men		
	N	Coef. (95% CI)	P value	N	Coef. (95% CI)	P value
**Maternal grandmother obesity grade**	**2844**			**763**		
Underweight	108	0.62 (-0.62, 1.45)	0.244	29	0.16 (-1.80, 2.11)	0.876
Normal weight	1778	Ref.		487	Ref.	
Overweight	596	0.38 (-0.12, 0.87)	0.136	148	-0.83 (-1.80, 0.14)	0.093
Obesity	362	1.21 (0.60, 1.81)	<0.001	99	1.26 (0.13, 2.39)	0.029
**Maternal grandfather obesity grade**	**2826**			**745**		
Underweight	144	0.33 (-0.58, 1.23)	0.480	30	-1.08 (-3.01, 0.86)	0.274
Normal weight	2133	Ref.		561	Ref.	
Overweight	409	0.68 (0.11, 1.24)	0.020	110	0.30 (-0.78, 1.38)	0.588
Obesity	140	1.81 (0.90, 2.73)	<0.001	44	1.95 (0.33, 3.57)	0.018
**Paternal grandmother obesity grade**	**2793**			**737**		
Underweight	104	1.10 (0.05, 2.16)	0.040	23	0.93 (-1.26, 3.13)	0.405
Normal weight	1749	Ref.		494	Ref.	
Overweight	588	0.57 (0.07, 1.07)	0.025	120	0.89 (-0.16, 1.94)	0.098
Obesity	352	1.77 (1.16, 2.38)	<0.001	100	1.31 (0.17, 2.44)	0.024
**Paternal grandfather obesity grade**	**2691**			**729**		
Underweight	164	0.77 (-0.08, 1.62)	0.076	28	-0.90 (-2.92, 1.11)	0.378
Normal weight	2025	Ref.		537	Ref.	
Overweight	345	0.30 (-0.31, 0.91)	0.336	105	0.72 (-0.39, 1.84)	0.203
Obesity	157	1.87 (1.00, 2.73)	<0.001	59	0.10 (-1.32, 1.53)	0.888
**Mother obesity grade**	**3271**			**864**		
Underweight	111	-0.21 (-1.23, 0.80)	0.681	19	-1.61 (-3.99, 0.77)	0.185
Normal weight	1608	Ref.		358	Ref.	
Overweight	1034	0.73 (0.32, 1.14)	0.001	316	-0.02 (-0.80, 0.76)	0.964
Obesity	518	2.21 (1.68, 2.73)	<0.001	171	1.14 (0.19, 2.08)	0.018
**Father obesity grade**	**3089**			**838**		
Underweight	123	0.42 (-0.55, 1.40)	0.395	23	3.10 (0.94, 5.26)	0.005
Normal weight	1760	Ref.		396	Ref.	
Overweight	784	0.64 (0.19, 1.09)	0.006	239	0.73 (-0.10, 1.56)	0.086
Obesity	422	1.89 (1.32, 2.45)	<0.001	180	2.15 (1.24, 3.06)	<0.001

Coefficient and 95% confidence intervals derived from linear regression models adjusted for age. For all comparisons, normal weight represents the reference category.

Figure S1. Associations between birthweight, size at birth, and personal life-course obesity grade with adult body mass index (BMI) in response to the intervention.

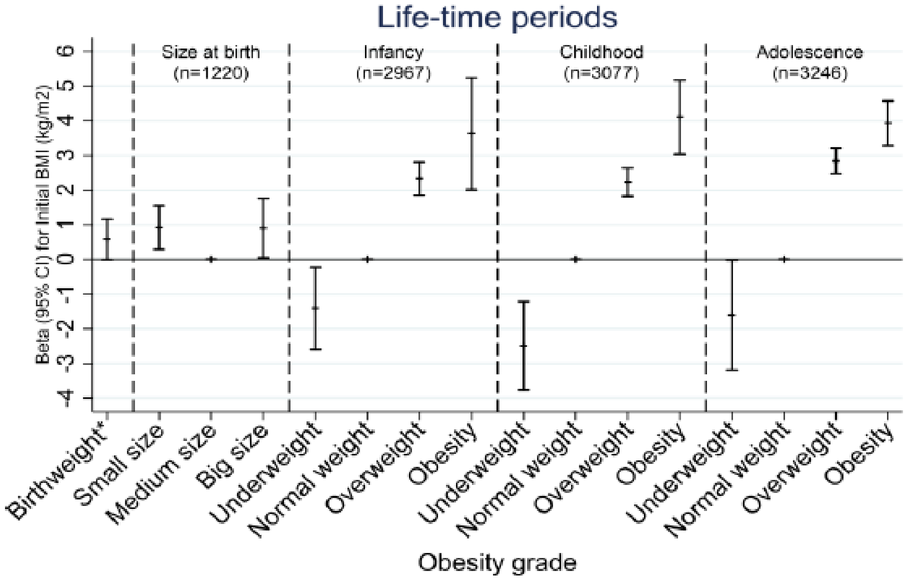

Lines represent beta coefficient and 95% CI derived from lineal regression models. All models adjusted for sex and age. *For birthweight, the coefficient represents the change in adulthood BMI (kg/m^2) as continuous per 1 kg increase in birthweight. For size at birth, medium size represents the reference category. For infancy, childhood and adolescence periods, normal weight represents the reference category.

Figure S2. The relation (and 95% confidence levels) of birth weight with body mass index (BMI) in adulthood. General additive models adjusted for sex and age. The symbols (+) on the X-axis indicate birth weight observations. Lines represent 95% confidence levels.

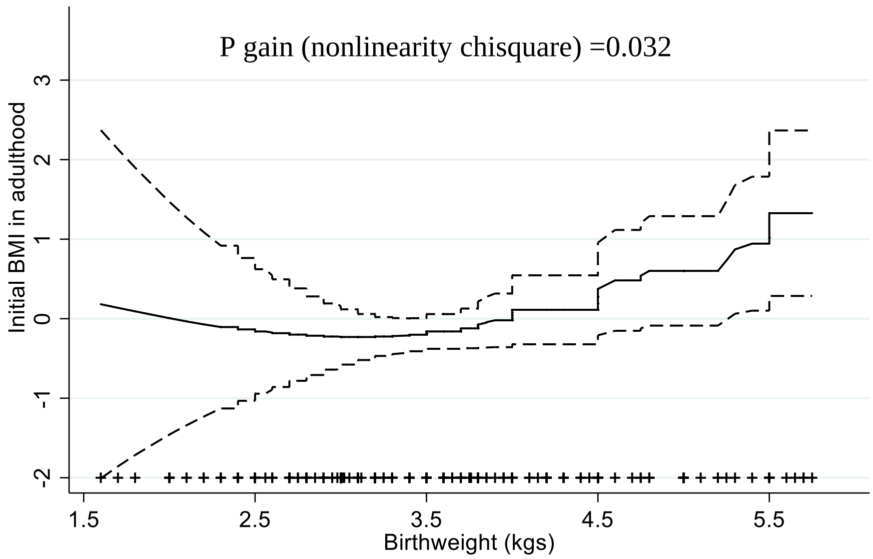

Table S8. Associations between birthweight, size at birth, and personal life-course obesity grade with body mass index (BMI) in adulthood stratified by sex.

	Women			Men		
	**N**	**Coef. (95% CI)**	**P value**	**N**	**Coef. (95% CI)**	**P value**
**Birth weight**	**583**	0.57 (-0.11, 1.25)	0.101	**170**	0.61 (-0.59, 1.80)	0.318
**Size at birth**	**935**			**285**		
Small	353	0.99 (0.29, 1.70)	0.006	87	0.65 (-0.73, 2.05)	0.356
Medium	473	Ref.		128	Ref.	
Big	109	1.05 (-0.01, 2.12)	0.053	70	0.56 (-0.93, 2.05)	0.457
**Infancy**	**2367**			**600**		
Underweight	61	-1.44 (-2.74, -0.15)	0.028	10	-1.55 (-4.60, 1.49)	0.316
Normal weight	1895	Ref.		450	Ref.	
Overweight	384	2.26 (1.70, 2.82)	<0.001	129	2.48 (1.52, 3.43)	<0.001
Obesity	27	5.06 (3.14, 6.99)	<0.001	11	0.006 (-2.89, 2.90)	0.997
**Childhood**	**2448**			**629**		
Underweight	53	-2.47 (-3.84, -1.09)	<0.001	9	-2.99 (-6.29, 0.31)	0.076
Normal weight	1665	Ref.		384	Ref.	
Overweight	662	2.40 (1.94, 2.86)	<0.001	212	1.55 (0.70, 2.39)	<0.001
Obesity	68	5.02 (3.79, 6.26)	<0.001	24	1.35 (-0.71, 3.41)	0.200
**Adolescence**	**2594**			**652**		
Underweight	32	-1.00 (-2.74, 0.74)	0.260	6	-4.84 (-8.75, -0.93)	0.015
Normal weight	1389	Ref.		359	Ref.	
Overweight	966	3.11 (2.70, 3.53)	<0.001	228	1.79 (0.97, 2.60)	<0.001
Obesity	207	4.00 (3.27, 4.74)	<0.001	59	3.61 (2.26, 4.96)	<0.001

Coefficient and 95% confidence intervals derived from linear regression models adjusted for age.
For comparisons, medium size at birth and normal weight represent the reference categories.

Table S9. Mediation analysis examining the mediated effect of maternal overweight-obesity on BMI in adulthood in the ONTIME study.

	Beta	CIL	CIU	P value
**Effect via birthweight (n=749)**				
ACME	0.51	-0.07	1.38	0.091
ADE	0.95	0.20	1.69	0.012
Total effect	1.46	0.47	2.56	0.002
Estimated proportion mediated	0.33	-0.09	0.76	0.091
**Effect via small size at birth (n=1032)**				
ACME	0.59	0.03	1.43	0.038
ADE	0.64	0.04	1.30	0.036
Total effect	1.23	0.39	2.15	0.001
Estimated proportion mediated	0.46	0.04	0.95	0.039
**Effect via big size at birth (n=774)**				
ACME	0.88	-0.07	2.27	0.073
ADE	1.08	0.34	1.81	0.005
Total effect	1.96	0.76	3.46	0.002
Estimated proportion mediated	0.44	-0.07	0.80	0.071
**Effect via infancy overweight-obesity (n=2863)**				
ACME	2.15	1.19	3.20	<2e-16
ADE	0.88	0.45	1.21	<2e-16
Total effect	2.99	1.97	4.07	<2e-16
Estimated proportion mediated	0.72	0.56	0.84	<2e-16
**Effect via childhood overweight- obesity (n=2984)**				
ACME	2.27	1.32	3.25	<2e-16
ADE	0.85	0.47	1.21	<2e-16
Total effect	3.11	2.09	4.12	<2e-16
Estimated proportion mediated	0.73	0.58	0.84	<2e-16
**Effect via adolescence overweight-obesity (n=3172)**				
ACME	2.94	1.85	4.01	<2e-16
ADE	0.83	0.49	1.18	<2e-16
Total effect	3.77	2.61	4.92	<2e-16
Estimated proportion mediated	0.78	0.66	0.87	<2e-16

ACME: average causal mediated effect; ADE: average direct effect (no mediation). Beta: effect size; CIL: 95% confidence Interval, lower limit; CIU: 95% confidence Interval, upper limit.

Table S10. Mediation analysis examining the mediated effect of paternal overweight-obesity on BMI in adulthood in the ONTIME study.

	**Beta**	**CIL**	**CIU**	**P value**
**Effect via birthweight (n=748)**				
ACME	0.63	-0.005	1.60	0.052
ADE	1.08	0.36	1.88	<2e-16
Total effect	1.72	0.69	2.98	<2e-16
Estimated proportion mediated	0.36	-0.005	0.70	0.052
**Effect via small size at birth (n=1027)**				
ACME	1.37	0.33	2.61	0.004
ADE	1.51	0.85	2.19	<2e-16
Total effect	2.87	1.66	4.28	<2e-16
Estimated proportion mediated	0.47	0.18	0.67	0.004
**Effect via big size at birth (n=769)**				
ACME	1.08	0.05	2.54	0.034
ADE	1.18	0.45	1.90	0.001
Total effect	2.26	0.89	3.91	<2e-16
Estimated proportion mediated	0.47	0.05	0.75	0.034
**Effect via infancy overweight-obesity (n=2676)**				
ACME	2.10	1.09	3.27	<2e-16
ADE	0.783	0.373	1.18	<2e-16
Total effect	2.88	1.77	4.04	<2e-16
Estimated proportion mediated	0.72	0.56	0.86	<2e-16
**Effect via childhood overweight-obesity (n=2795)**				
ACME	2.07	1.08	3.16	<2e-16
ADE	0.69	0.28	1.07	<2e-16
Total effect	2.76	1.69	3.85	<2e-16
Estimated proportion mediated	0.75	0.58	0.89	<2e-16
**Effect via adolescence overweight- obesity n=2977**				
ACME	3.00	1.78	4.24	<2e-16
ADE	0.77	0.41	1.13	<2e-16
Total effect	3.77	2.51	5.4	<2e-16
Estimated proportion mediated	0.79	0.67	0.89	<2e-16

ACME: average causal mediated effect; ADE: average direct effect (no mediation). Beta: effect size; CIL: 95% confidence Interval, lower limit; CIU: 95% confidence Interval, upper limit.
